# Assessment of the Risk of Microplastics on Gill and Gut Health and Subsequent Pathogen Susceptibility in the Goldfish Model

**DOI:** 10.4014/jmb.2504.04019

**Published:** 2025-08-18

**Authors:** Ho Sung Kim, Bohyun Yun, Yongjoon Yoon, Jeong Woo Park, Jimin Hyun, BoMi Ryu, Aaron M. Yerke, Sungmin Hwang, Ki Hwan Moon

**Affiliations:** 1 Department of Convergence Interdisciplinary Education of Maritime & Ocean Contents, Korea Maritime & Ocean University, Busan 49112, Republic of Korea; 2 Division of Convergence on Marine Science, Korea Maritime & Ocean University, Busan 49112, Republic of Korea; 3 Division of Practical Research, Honam National Institute of Biological Resources, Mokpo-si 58762, Republic of Korea; 4 Major of Food Science and Nutrition, Pukyong National University, Busan 48513, Republic of Korea; 5 Department of Bioinformatics and Genomics, University of North Carolina at Charlotte, Charlotte, NC 28223, USA

**Keywords:** Microplastic, gill & gut health, goldfish, immune/stress-responses, pathogen susceptibility

## Abstract

Microplastics are pervasive pollutants in aquatic ecosystems, yet their effects on fish tissues remain insufficiently characterized. Our study investigates the impact of polystyrene microplastics (0.5 and 2 μm) on the gill and intestinal tissues of goldfish (*Carassius auratus*), with a focus on inflammatory responses and pathogen susceptibility. Following two weeks of exposure, histological and molecular analyses revealed increased filament cartilage thickness in gills, enhanced villus thickness and goblet cell numbers in intestines, and upregulation of immune- and oxidative stress-related genes. Exposure to 0.5 μm microplastics significantly reduced survival after *Edwardsiella piscicida* infection, indicating increased vulnerability to pathogens. These findings highlight the immunotoxic effects of microplastics and their potential to compromise fish health in contaminated environments.

## Introduction

As energy demand gradually increases due to population growth and industrial development, the generation of environmental pollutants is also increasing. Environmental pollution resulting from this has recently drawn public attention [1-3]. Pollutants generated on land move toward the aquatic environment through water flow [4]. Various land-based pollutants can flow into the aquatic environment and have fatal effects on aquatic organisms such as fish, shellfish, and mollusks [5]. Unlike terrestrial environments, aquatic environments are in constant contact with the surface of aquatic organisms, so these pollutants can be more dangerous against living organisms [2].

Plastic is synthetic chemical mainly derived primarily from petroleum, composed mainly of hydrocarbon chains [6]. Compared to other materials, plastic has various advantages such as low price, light weight, chemical stability, and ease of processing, making them widely used in many fields [7]. Global plastic production has steadily increased and is projected to continue rising, leading to an expected growth in plastic waste in the future. Gil-Jasso reported that production of microplastics were expected to reach 1.12 million tons by 2050 [8]. However, only a small fraction of plastic waste is recycled, while the majority is either landfilled or incinerated, raising concerns about the high potential for plastic waste to leak into the environment [9-11]. According to previous reports, more than 9 million metric tons of plastic waste enter the ocean each year, and the United Nations (UN) estimates that between 75 and 199 million metric tons of plastic are currently floating in the world's oceans [12, 13]. The biggest problem with plastic waste introduced into the environment is the recalcitrance of degradation. These plastic wastes are broken down into small pieces by physical, chemical, and biological factors and remain in the form of small particles, called microplastics [10].

Microplastics are plastic particles with a size smaller than 5 mm, and can be divided into primary microplastics and secondary microplastics depending on the production process [14-16]. Primary microplastics are small plastic particles produced for a specific purpose, and are mainly in the form of beads. Secondary microplastics refer to plastic particles that have been broken into small pieces by various factors, and mainly in the form of fibers.

Microplastics released into aquatic environments can be absorbed by their inhabitants, causing various harmful effects. Previous studies have shown that microplastic exposure increases stress, alters global gene expression, and impacts factors involved in diet, growth, and development in aquatic species [17-19]. Additionally, transport of microplastics through the food chain to higher trophic levels increases the risks of bioaccumulation and potential disruption of physiological functions in larger organisms including humans [20].

For this project, Goldfish (*Carassius auratus*) were chosen as the model organism due to their suitability as a small, non-mammalian animal model, their adaptability to laboratory conditions, low cost, and ease of handling. Goldfish offer stable genetic tractability, large clutch sizes, and are highly amenable to studies involving cell biology and molecular genetic engineering [21, 22]. Additionally, goldfish have been widely used in ecotoxicological studies as an appropriate model organism for assessing the toxicity of microplastics [23-25].

Previously, we confirmed that polystyrene nanoplastics can affect to the pathogenicity of the fish pathogen *Edwardsiella piscicida* [26]. Therefore, this study aims to verify the effects of microplastics accumulation on the host, and to evaluate the changes in the host's immunity and pathogen susceptibility due to microplastic accumulation. Despite the growing environmental pollution from microplastics, including various types such as polystyrene, polyethylene, and polypropylene, the specific effects of polystyrene microplastic accumulation on gill tissue remain underexplored. To address this gap, our research also focuses on examining how exposure to polystyrene particles (0.5 and 2 μm) influences inflammation and immune responses in fish gills. In particular, this study investigated the penetration and retention of microplastics of two distinct sizes within the goldfish host, and examined how these size-dependent differences impact the host.

## Materials and Methods

### Experimental Animals

Goldfish (*C. auratus*) were purchased from a commercial aquarium, Gwang-won aquarium (Republic of Korea). The average weight and length of the goldfish used in this study were 3.18 ± 0.21 g and 6.84 ± 0.17 cm, respectively. Goldfish were acclimatized for a week in the Aquatic Eco-Systems (21 Century High Tech, Republic of Korea). Photoperiod was maintained at 14:10-h light/dark. The breeding water was filtered by reverse osmosis and water parameters were monitored to maintain water temperature 23 ± 1°C, pH 7.5 ± 0.2, and conductivity 652 ± 50 μS/cm. The fish were fed with a commercial diet (PRODAC, Italy) twice per day, at 10 AM and 6 PM.

### Microplastic Exposure Assay

The microplastics were purchased from Bangs Laboratories (USA) and polystyrene beads of sizes 0.5 and 2 μm were used in the exposure assay.

Three glass tanks (45 cm × 30 cm × 31 cm) were used with 20 L of Aquatic Eco-Systems water, and each tank was used as non-treated (control), 0.5 μm microplastics exposure group (0.5 μm), and 2 μm microplastics exposure group (2 μm), respectively. The concentrations of 0.5 and 2 μm microplastics for exposure assay were 100 μg/l (2.05 × 10^4^ beads/ml and 1.35 × 10^6^ beads/ml, respectively). A total of 120 goldfish was exposed to each of the three treatments for 14 days, so that each assay could be conducted in triplicate. The tank water was refreshed every three days by discarding 10 L of the old breeding water and adding 10 L of the new breeding water and microplastics were additionally added to reach the same concentration as the old breeding water. During the exposure assay, fish were fed a commercial diet twice per day. Fish were fasted for at least 12 h before the end of the exposure assay.

### Microplastics Bioaccumulation Analysis

After 2 days of microplastic exposure, the accumulation of microplastics in tissues was analyzed using three goldfish per group, following the method modified from Kim *et al*. [25]. Briefly, goldfish tissue samples were digested in a 10% potassium hydroxide (KOH) solution at 60°C for 48 h. Microplastics of 0.5 μm and 2 μm were then isolated using a 0.2 μm filter. The separated microplastics were further treated with a zinc chloride solution (density: 1.8 g/cm³) for 24 h. The supernatant containing microplastics was filtered using a vacuum pump through a polycarbonate track-etched membrane filter (diameter: 47 mm, pore size: 0.1 μm; GVS, Italy). The filters were then placed in petri dishes and dried at room temperature for 24 h. Subsequently, the filters were stained with 200 μl of Nile Red solution (5 mg/l) and incubated in the dark for 30 min. After staining, the filters were rinsed with 100 μl of *n*-hexane and analyzed using a KI-3000 fluorescence microscope (KoreaLabTech., Republic of Korea) to quantify the microplastic particles. Microplastics stained with Nile red exhibited fluorescence with an excitation peak at 465–495 nm and an emission peak at 515–555 nm. Image analysis was performed using OptiView software v.4.11 (KoreaLabTech).

### Histological Analysis

Three fish per group were randomly selected and euthanized using tricaine methane sulfonate (Sigma-Aldrich, USA). Gill and intestine tissues of goldfish were sectioned and fixed by using Dietrich’s fixative [27]. The fixed gill and intestine tissues were dehydrated in ethanol, hyalinized in xylene, embedded in paraffin wax, and sectioned into 5 μm thickness. The paraffin sections were stained with hematoxylin and eosin (H&E). Stained sections were observed with an optical microscope. Filamentous cartilage and villus thickness, and the number of goblet cells were evaluated as biomarkers of gill and intestinal inflammation. The thickness of filament cartilage and villus were calculated by OptiView software v.4.11, and the average number of goblet cells per villus was calculated for statistical analysis.

### Quantitative Reverse Transcription-Polymerase Chain Reaction (qRT-PCR)

Total RNA of goldfish liver was extracted using TRIzol Reagent (Thermo Fisher Scientific, USA) according to the product instruction. An additional DNA digestion step was conducted using a Turbo DNA-free kit (Invitrogen, USA). The DNA-free RNA was confirmed by end-point PCR with 35 cycles, targeting the endogenous control gene β-actin. 2X OneStep qRT-PCR Master Mix (Biofact, Republic of Korea) was used to amplify the target genes using a QuantStudio 1 Real-Time PCR System (Applied Biosystems, USA). Each primer set is listed in Table S1. The specificity of the primer sets was examined by a melting curve. The expression levels of target genes were normalized to the endogenous control gene β-actin, and the relative expression of target genes was calculated by Livak’s method [28].

### Enzyme Activity Assay

Enzyme activity was measured using a Fish Lysozyme ELISA kit, a Fish Alkaline Phosphatase ELISA Kit, a Fish Acid Phosphatase ELISA kit, and a Fish superoxide dismutase ELISA kit (MyBioSource, USA) and all assays were performed in triplicate according to the manufacturer’s instructions. Serum samples were collected from three goldfish in each group (control, 0.5 μm, and 2 μm), and the activity of all enzymes was measured by absorbance at 450 nm wavelength with SYNERGY H1 microplate reader (BioTek, USA).

### Survival Assay

Goldfish were anesthetized with tricaine methane sulfonate before intraperitoneal (IP) injection. *E. piscicida* CK41 strain was cultured in Luria-Bertani (LB) broth (Gibco, USA) at 27°C with shaking at 200 rpm until the optical density at 600 nm (OD_600_) reached 0.6. Bacterial cells were harvested by centrifugation at 2,000 ×*g* for 10 min, and washed three times with phosphate buffered saline (PBS). Diluted bacterial cells (100 μl) were injected in the abdominal cavity of 10 anesthetized goldfish and divided into three conditions (control, 0.5 μm, and 2 μm). All fish were monitored for 30 days post-injection, and mortalities were recorded every day.

### Statistical Analysis

All statistical analyses used in this study were performed by GraphPad Prism 9 v.9.5.1 (GraphPad, USA). The statistical significance was analyzed using one-way ANOVA. The qRT-PCR data were analyzed using a two-way ANOVA, and Dunnett's multiple comparison test was applied to compare each group (0.5 μm and 2 μm) to the control group. Statistical significance was indicated by asterisks: * indicates *p* < 0.05, ** indicates *p* < 0.01, *** indicates *p* < 0.001, and **** indicates *p* < 0.0001.

### Ethical Statement

This study was approved by the National Korea Maritime & Ocean University-Institutional Animal Care and Use Committee (KMOU-IACUC), Busan, Republic of Korea (Approval Number: KMOU IACUC 2023-01). All of the experimental procedures were conducted in accordance with the Institutional Animal Care guidelines of Korea Maritime & Ocean University.

## Results

### Polystyrene Microplastic Beads Accumulation in Gill and Intestinal Tissues

**Fig. 1:**
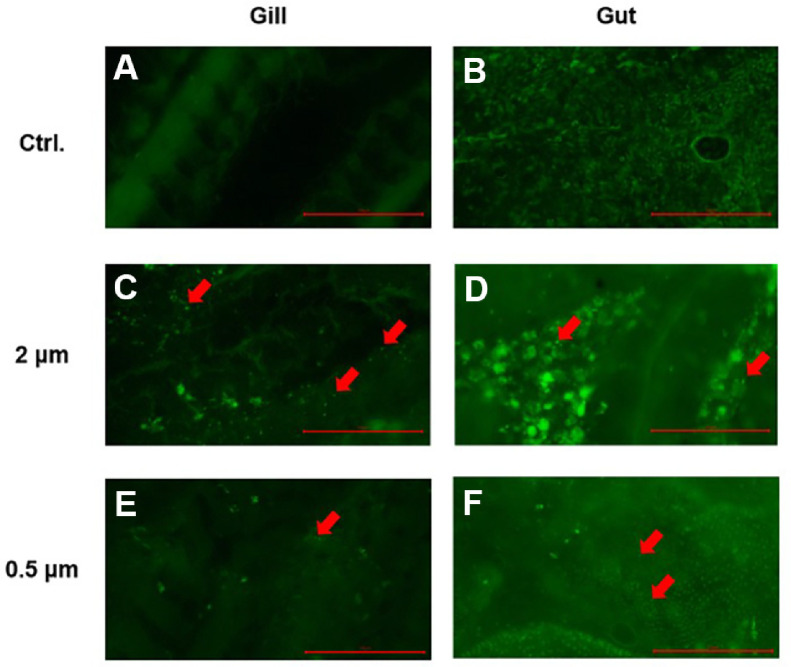
Microplastic accumulation detected in the gill and intestine tissues in goldfish. Representative images show the accumulation of fluorescent 0.5 μm, and 2 μm of polystyrene beads in both gill and intestine tissues. Red arrows indicate microplastics. Scale bar: 100 μm.

**Fig. 2:**
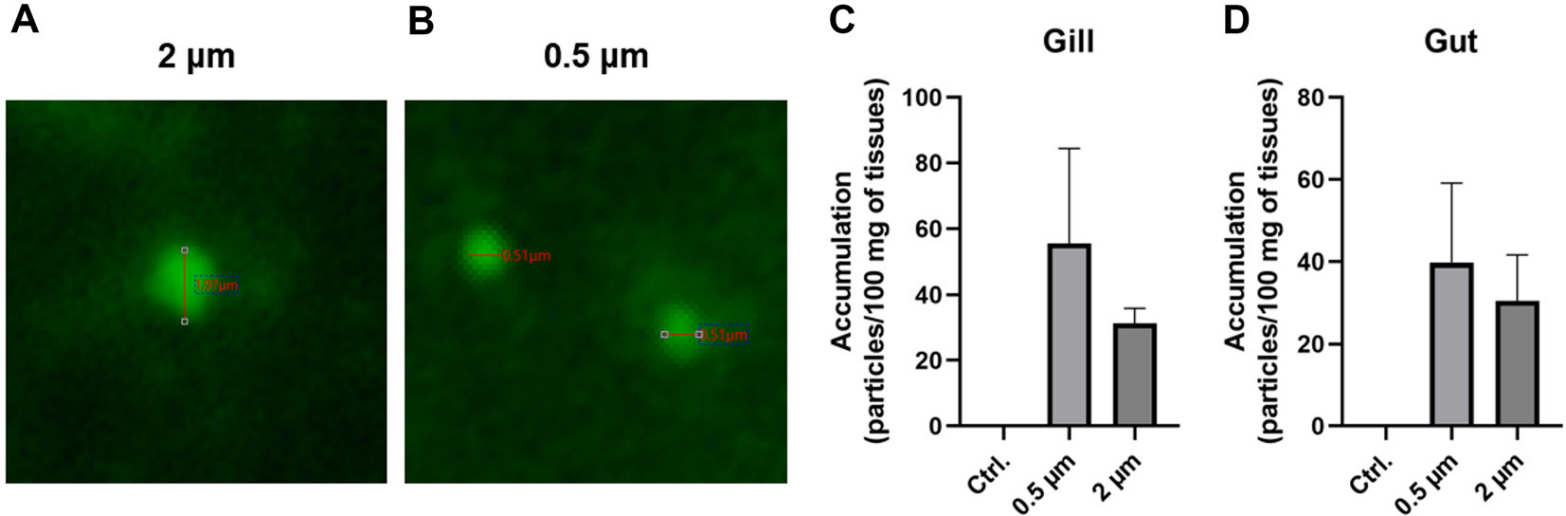
Microplastic accumulation detected in the filter. Representative images show the presence of fluorescent 0.5 μm (**A**) and 2 μm (**B**) of polystyrene beads in the filter. Bar plots show the differences in the number of microplastics in gill (**C**) and gut tissues (**D**) for each treatment.

To evaluate the bioaccumulation of microplastics on the fish host, goldfish were exposed to 0.5 and 2 μm polystyrene microplastic beads, following the methods described in the section 2.2. After 2 days, the goldfish were euthanized, and their gill and intestine samples were exercised to observe the accumulation of microplastic beads. We confirmed the presence of fluorescence in the tissues. The visualizations revealed fluorescence in both 0.5 μm and 2 μm microplastics (Fig. 1). To identify and quantify the accumulation of microplastics, tissue samples were processed using the filtration method described above. The presence of microplastics was also confirmed in the filter (Fig. 2A and 2B). In the gill tissue, the number of microplastic particles per 100 mg of tissue was 31.32 ± 4.51 for 2 μm particles and 55.42 ± 29.07 for 0.5 μm particles. In the gut tissue, the number of microplastic particles per 100 mg of tissue was 30.48 ± 11.19 for 2 μm particles and 39.78 ± 19.30 for 0.5 μm particles.

### Increase Inflammatory Response in Gill and Intestinal tissues Induced by Physical Stimulation of Microplastics

**Fig. 3:**
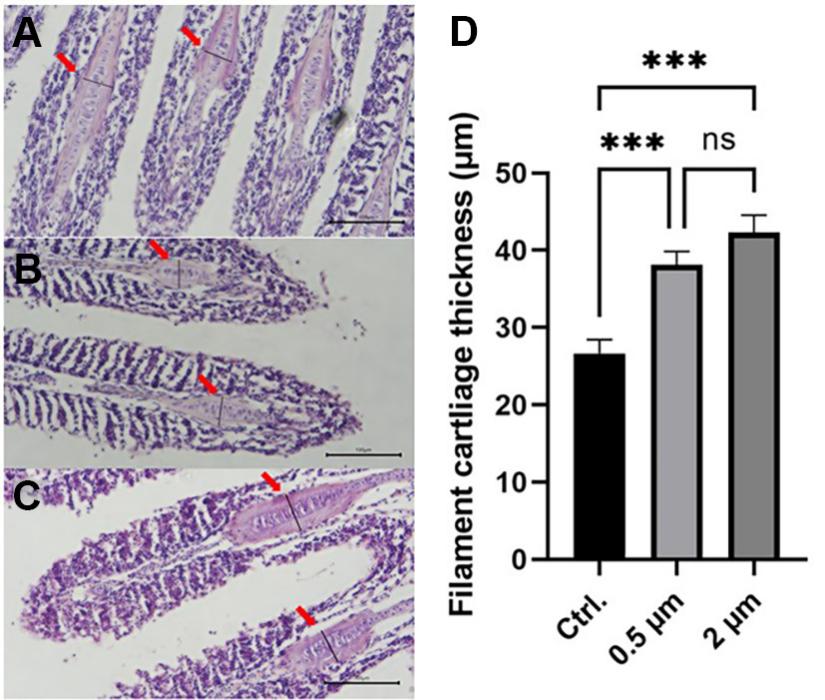
Histopathological analysis of filament cartilage in goldfish gills. Gill transversal paraffin sections were stained with hematoxylin and eosin show levels of filament cartilage from representatives of the control (**A**) 0.5 μm (**B**) and 2 μm (**C**) groups. Red arrows indicate the filament cartilage, while the black bar denotes the location where the measurement was taken. The black scale bar represents 100 μm. Bar plots (**D**) show filament cartilage thickness where asterisks indicate significant differences between experimental and control groups as determined by one-way ANOVA with *** indicating *p* < 0.001.

**Fig. 4:**
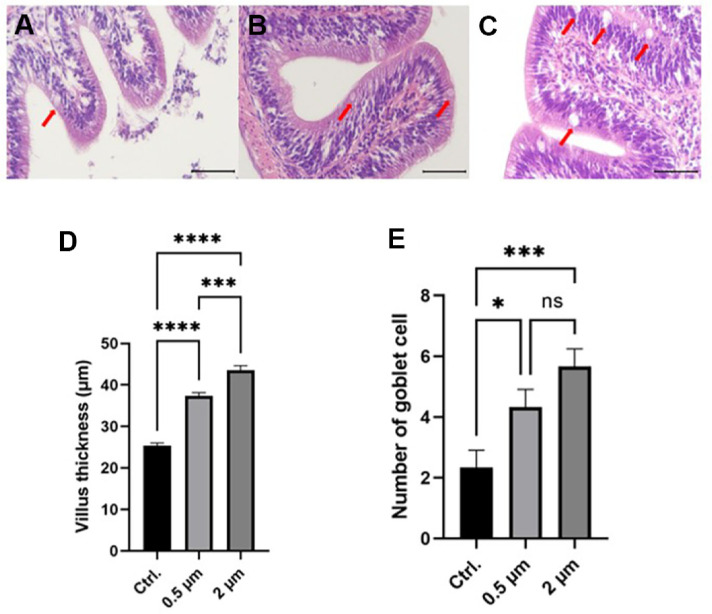
Histopathological analysis of intestine tissues in goldfish. Intestine transversal paraffine sections stained with hematoxylin and eosin show villus thickness and number of goblet cells in representatives from the control (**A**) 0.5 μm (**B**) and 2 μm (**C**) treatments. Red arrows indicate goblet cells. The black scale bar represents a distance of 50 μm. Bar plots show and villus thickness (**D**) and number of goblet cells (**E**). Asterisks indicate significant difference as determined by one-way ANOVA (** indicates *p* < 0.01, *** indicates *p* < 0.001, and **** indicates *p* < 0.0001).

We performed H&E staining to confirm the inflammatory response caused by exposure to microplastics. Gill and intestinal tissues of goldfish exposed to microplastics generally showed increased inflammatory response (Fig. 3 and 4). In gill tissue, microplastics exposure significantly increased the thickness of filament cartilage (Fig. 3). In intestinal tissue, similar to gill tissue, villus thickness and number of goblet cells, which are indicators of immune response in intestinal tissue, were significantly increased in the 0.5 and 2 μm groups (Fig. 4).

### Gene Expression of Immune and Oxidative Stress-Related Genes Induction by Microplastics Exposure

**Fig. 5:**
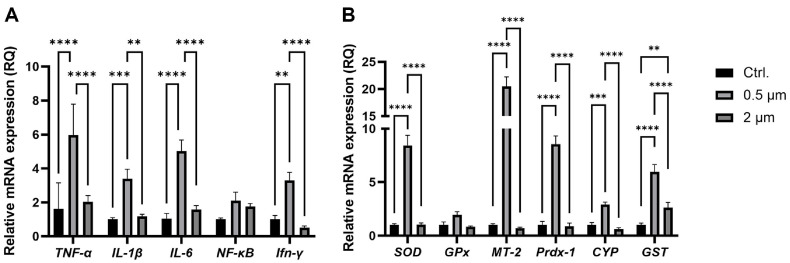
Transcriptional levels of immune/oxidative stress-related genes expression. Bar plots show immunerelated gene expression (**A**) and oxidative stress-related gene expression (**B**). Expression levels of selected genes in qRT-PCR were quantified by comparison with internal control genes of β-actin. Error bars represent the standard deviation of three biological replicates. Asterisks indicate significant differences between treatments as determined by two-way ANOVA (** indicates *p* < 0.01, *** indicates *p* < 0.001, and **** indicates *p* < 0.0001).

qRT-PCR was conducted to confirm the increased tissue inflammatory response caused by microplastics exposure at the transcriptional level. Tumor necrosis factor alpha (TNF-α), interleukin 1 beta (IL-1β), interleukin 6 (IL-6), nuclear factor kappa B (NF-κB), and interferon gamma (IFN-γ) were used as immune response markers, and superoxide dismutase (SOD), glutathione peroxidase (GPx), metallothionein 2 (MT-2), peroxiredoxin 1 (Prdx-1), cytochrome P450 (CYP), and glutathione S-transferase (GST) were selected as biomarkers of oxidative stress response [29, 30]. Compared to the group of 2 μm, qRT-PCR results showed that the expression levels of five immune-related genes and six oxidative stress-related genes were increased in the 0.5 μm group (Fig. 5). These results indicate that exposure to microplastics with a relatively small size of 0.5 μm induced a stronger inflammatory response than 2 μm microplastics in the goldfish at the molecular level.

### Increased Activity of Immune and Oxidative Stress-Related Enzymes after Microplastics Exposure

**Fig. 6:**
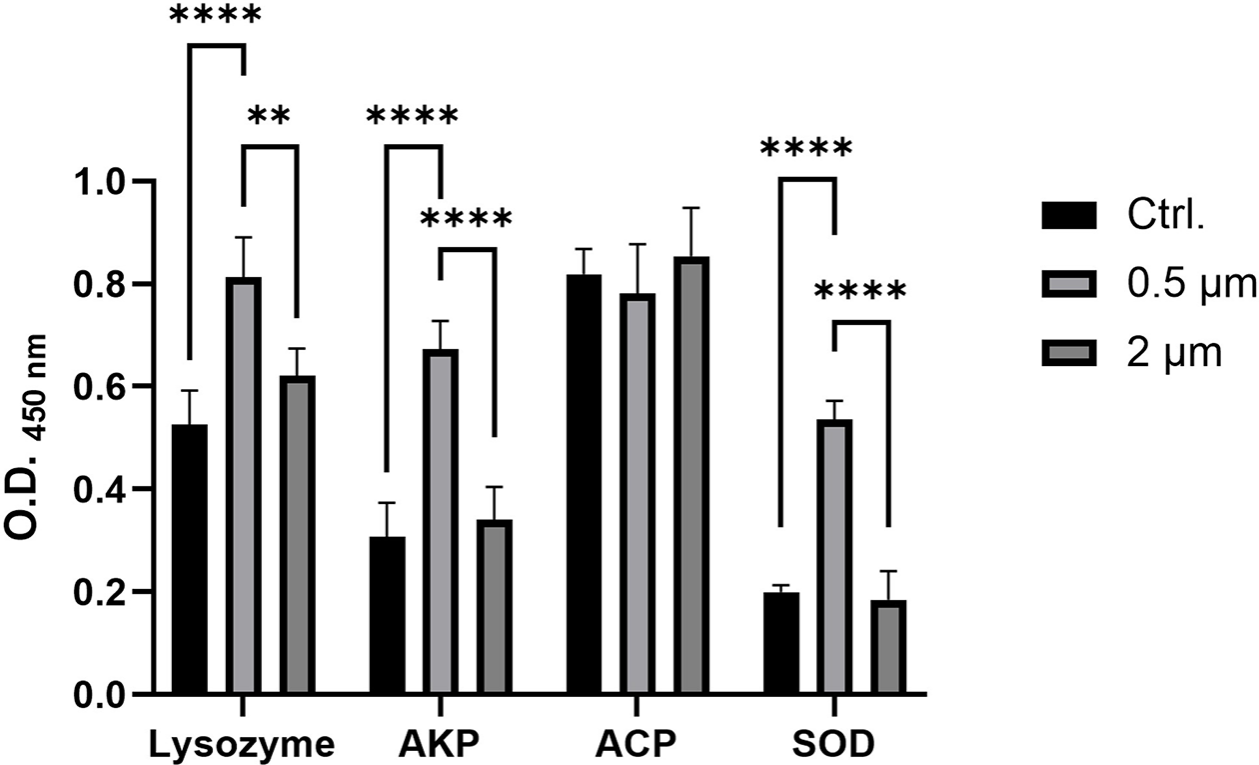
Analysis of immune/oxidative stress-related enzymes expression. Expression levels of lysozyme, an immune-related enzyme, and alkaline phosphatase (AKP), acid phosphatase (ACP), and superoxide dismutase (SOD), which are oxidative stress-related enzymes, were confirmed by measuring absorbance. Error bars represent the standard deviation of three biological replicates. Asterisks indicate significant differences between treatments as determined by two-way ANOVA (** indicates *p* < 0.01, *** indicates *p* < 0.001, and **** indicates *p* < 0.0001).

Enzyme activity assay was conducted to confirm the effect of microplastic exposure on enzyme expression levels within the host. Lysozyme was reported as a marker related to immune response and alkaline phosphatase, acid phosphatase, and superoxide dismutase (AKP, ACP, and SOD, respectively) were reported as markers of oxidative stress [31, 32]. Enzyme activity assay results showed that lysozyme, AKP, and SOD expression were significantly higher in the 0.5 μm group compared to the 2 μm group (Fig. 6).

### Alteration of Goldfish Pathogen Susceptibility after Exposure to Microplastics

**Fig. 7:**
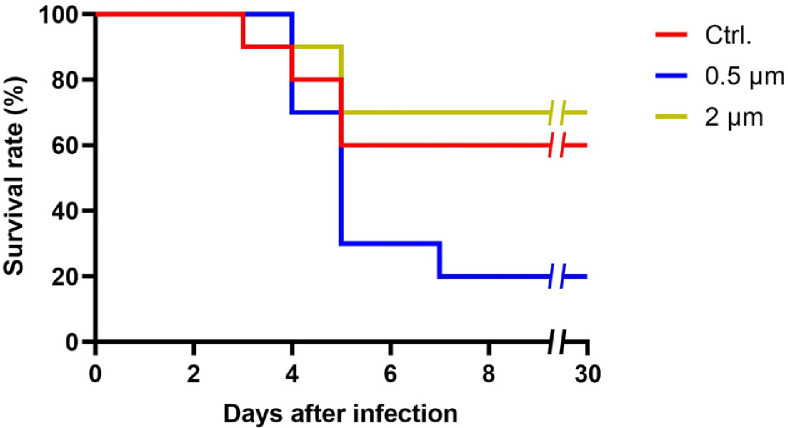
Confirmation of the goldfish’s pathogen susceptibility by *E. piscicida* CK41 injection. A plot of the survival rate of the goldfish from each treatment after injection with 3.4 × 10^5^ CFUs of *E. piscicida* CK41 per goldfish (*n* = 10 per group) shows the effect of microplastic size on pathogen susceptibility. The survival rate of goldfish was monitored for 30 days.

*E. piscicida*, a Gram-negative bacteria causing Edwardsiellosis, is a representative fish pathogen causing enormous economic losses for the aquaculture industry [33]. To confirm the effect of microplastics exposure on host pathogen susceptibility, we performed a survival assay by injecting *E. piscicida* CK41 into the abdominal cavity of the goldfish. All groups were injected with 3.4 × 10^5^ CFUs of *E. piscicida* CK41 and monitored for 30 days. The survival rate for 30 days after *E. piscicida* CK41 injection is shown in Fig. 7. The 2 μm group showed a survival rate similar to the control group, whereas the 0.5 μm group showed a significantly reduced survival rate (Fig. 7). This result, similar to those confirmed at the transcriptional and translational levels, implies that exposure to 0.5 μm microplastics has negative effects on the host in addition to increasing pathogen susceptibility.

## Discussion

Microplastics, a representative aquatic environmental pollutant, are hard to decompose and remain in the environment for long periods of time, posing a risk of exposure to aquatic organisms [10, 11]. Microplastics affect the ecosystem not only as a physical irritant but as a means of transport for various pollutants and microorganisms [34, 35]. Currently, the environment is polluted by various types of microplastics. Polystyrene microplastics are a significant environmental pollutant, accounting for approximately one-quarter of plastic debris found in marine environments [36]. Additionally, due to the presence of a benzene ring in its molecular structure, polystyrene exhibits a high affinity for binding with various other environmental pollutants [37, 38]. In previous studies, various sizes of microplastics accumulated in the gill and gut, inducing oxidative stress and immune responses in fish models [23, 39]. Therefore, we hypothesized that inflammatory response and oxidative stress in the host induced by microplastics would affect the host’s pathogen susceptibility. In this study, we exposed goldfish to polystyrene particles of 0.5 and 2 μm in size for two weeks.

Fluorescence detection confirmed the presence of microplastics in the gill and intestinal tissues, though non-specific fluorescence signals were also observed (Fig. 1). To validate that the detected fluorescence originated from microplastics, tissue digestion and membrane filtration were performed, revealing microplastic particles matching the size of those used in this study (Fig. 2A and 2B). These findings indicate that microplastics can accumulate in both gill and intestinal tissues of goldfish. Previous studies have shown that polystyrene microplastics can accumulate in the various tissues of fish, and the accumulation levels of microplastics within fish vary depending on the exposure duration, concentration, and size of the microplastics [23, 40, 41]. Similarly, although not statistically significant, we observed a trend indicating that smaller microplastics and a higher number of beads lead to increased accumulation (Fig. 2C and 2D). These findings confirm that microplastics can accumulate in the tissues of goldfish.

The results of H&E staining performed on the gill and intestinal tissue indicate that exposure to microplastics induced an inflammatory response in the goldfish tissues (Figs. 3 and 4). The thickness of filament cartilage in the gill tissue is an indicator used to confirm the host’s health status and inflammatory response [42]. The thickness of filament cartilage significantly increased in the gill tissue of Nile tilapia infected with parasites, and the thickness of filament cartilage returned to the normal range when the disease was alleviated [43]. In the case of intestinal tissue, the thickness of villus and the number of goblet cells, which are widely known as indicators of inflammatory response [44], were significantly increased in the group exposed to microplastics. This inflammatory response is presumed to result from the physical stimulation of the tissue surface by microplastics, which intensifies as the surface roughness of the particles increases. Overall, the 2 μm microplastics exhibited a tendency to induce slightly greater histopathological effects in goldfish, although some differences were not statistically significant. This observation is consistent with previous studies suggesting that larger microplastics are generally more likely to cause tissue damage [45].

qRT-PCR analysis revealed upregulation of immune and oxidative stress-related genes in the 0.5 μm microplastic exposure group (Fig. 5). However, most genes showed no change in expression level or were downregulated in 2 μm microplastic exposure group (Fig. 5). This outcome contradicts previous histological analysis findings and is likely attributed to the physical characteristics of the microplastic. Large microplastics enter the digestive tract and are mostly excreted out of the body through feces [46]. However, smaller microplastics are more likely to remain in the body because their small size allows them to become embedded in narrow anatomical structures, such as the folds of the intestinal villi, preventing excretion [47]. For this reason, large microplastics can cause greater damage to tissues, but small microplastics have the potential to cause continuous irritation to the host. Enzyme activity assays to confirm the expression levels of immune and oxidative stress related enzymes also showed similar results to qRT-PCR. In the 0.5 μm microplastic exposure group, lysozyme, AKP, and SOD, excluding ACP, all showed higher expression levels compared to the control (Fig. 6). The increase in the expression level of enzymes was expected as a result of inflammatory response induced by exposure to 0.5 μm microplastics. The increase in the lysozyme expression is expected due to the invasion of various antigen into wounds caused by physical stimulation of microplastics and an increase in phagocytosis to defend against these antigens [48]. SOD is an enzyme responsible for eliminating reactive oxygen species (ROS), and its expression levels appear to have increased in response to the elevated ROS generated by the immune reaction [32]. AKP is a non-specific biomarker for evaluated the liver and bone damage, and cellular stress, and plays an essential role in providing energy to the stressed cell [49]. In the 0.5 μm microplastic exposure group, the increase in serum AKP might be related to the increase in the expression of immune and stress genes in the liver as confirmed by qRT-PCR, suggesting the possibility that 0.5 μm microplastic can accumulate in tissues and exhibit cytotoxicity for more than 2 weeks. Notably, among the four enzymes used as oxidative stress markers in this study (lysozyme, AKP, SOD, and ACP), ACP did not exhibit significant differences in either the 0.5 μm or 2 μm microplastic exposure groups compared to the control. While the exact reason remains unclear, it is possible that this result reflects the nature of ACP's role in cellular processes. ACP is an essential lysosomal enzyme and a biomarker of nonspecific immune responses in fish models [50-52]. In previous studies, necrosis or apoptosis was induced by cytotoxic agents, leading to increased ACP levels in tissues [50, 53]. In contrast, our results show no significant differences in serum ACP levels compared to the control group, which may be attributed to tissue-specific characteristics. Therefore, it is plausible that ACP does not exhibit a measurable response to microplastic exposure, in contrast to the other markers. Through our experiments, we have shown that microplastic exposure increases the host’s inflammatory response and stress and causes an altered the host immune responses that directly affects gill and gut health in fish host.

To assess the impact of microplastics on the host's susceptibility to pathogens, *E. piscicida* CK41 was IP injected into goldfish, and their survival rates were monitored for 10 days. The survival rate of the 2 μm microplastics exposure group was not significantly different from the control group, while the 0.5 μm microplastics exposure group showed a significantly lower survival rate (Fig. 7). The lower survival rate of the 0.5 μm microplastics exposure goldfish may be due to the regulation of immune and oxidative stress factor regulated by 0.5 μm microplastics, which is confirmed in the qRT-PCR and ELISA assays (Figs. 5 and 6). This suggests that exposure to microplastics can eventually increase susceptibility to pathogens, which can have a negative effect on the host, easily leading to death even when exposed to a small number of pathogens.

However, the reason for the nonsignificant immune response and survival rate difference in the 2 μm microplastic exposure group is still unclear. We presume that this occurred due to the difference in cellular internalization according to the size of the microplastic. In a recent study, exposure of rainbow trout-derived epithelial cells and fibroblasts to microplastics revealed that while 2–5 μm microplastics exhibited limited cellular uptake, 1 μm microplastics were efficiently internalized via macropinocytosis and endocytosis, whereas larger particles (2–5 μm) were more likely to weaken the epithelial barrier and translocate into underlying tissues [54]. Additionally, it was reported that 0.5 μm size polystyrene microplastic showed higher bioavailability, cellular uptake, and oxidative stress induction than 5 μm size polystyrene microplastic, resulting in more prominent toxic effects *in vitro* and *in vivo* [55]. These findings suggest that 0.5 μm microplastics may exhibit greater toxicity than 2 μm microplastics. However, 2 μm polystyrene has also been reported to induce changes in gut microbiota, leading to behavioral alterations, or act as a vector for toxic substances [56, 57].

This study investigates the risks of microplastic exposure, focusing on how microplastics induce inflammatory responses and stress, compromising gill and gut health and increasing susceptibility to pathogens in aquatic organisms. Furthermore, in our knowledge, this is the first study that finds that microplastic exposure in goldfish may influence the immune and oxidative stress, leading to increased susceptibility to *E. piscicida*. Our findings provide scientific evidence of the harmful impact of microplastic pollution in aquatic environments and emphasize the need for effective plastic waste disposal systems to prevent further environmental contamination.

## Supplemental Materials

Supplementary data for this paper are available on-line only at http://jmb.or.kr.
