# Supplementary material for: Assessment of the Risk of Microplastics on Gill and Gut Health and Subsequent Pathogen Susceptibility in the Goldfish Model
Source: J Microbiol Biotechnol. 2025 Aug 18;35:e2504019. doi: 10.4014/jmb.2504.04019 (PMC12375542; doi:10.4014/jmb.2504.04019)
Supplement: Supplementary file 1 [file jmb-35-e2504019-supple.pdf]

J. Microbiol. Biotechnol. 2025. 35: e2504019
https://doi.org/10.4014/jmb.2504.04019

Table S1. Primers used in this study

Primer	Sequence (5'-3')	Amplicon size (bp)
β-actin_F<br>β-actin_R	CAG GTA TGG AGT CTT GCG GT<br>CAG AGC CTA GCT CAC CTT GAT	200
Tnf-α_F<br>Tnf-α_R	ATG CGG CTC TAC TTG AAC GG<br>GCT AGG AGA CAA CAC AGA TGT TCA G	188
IL-1β_F<br>IL-1β_R	GAA GGT CCG TGC ACC ACA A<br>GTT GTA AGC TGT GCC CGT CT	186
IL-6_F<br>IL-6_R	TTC GTG TCT GGG TCG CAT TT<br>CTG AGG TGT GTT TAC CTG GCT	157
NF-κB_F<br>NF-κB_R	TTG CGA ATC CAA AGG GGA CA<br>TCT GTG ATG ACG GCG AGA TG	196
Ifn-γ_F<br>Ifn-γ_R	TTG CAT CAT CTT GCC CTT GAA CTT<br>GGG CAC AGC TCA TTT ATC TTC AAA G	150
SOD_F<br>SOD_R	ACA CGT CGG AGA CCT TGG TA<br>ACA CCA TGA GGT CAG CTG GTA	181
GPx_F<br>GPx_R	GAC TCC GTG TCC TTG ATG GG<br>TGC CAG GTT TAT TTC GCC CTC	197
MT-2_F<br>MT-2_R	GAG TAC GTG AAC CAC AAC CCA<br>GGA ATT GCC CTT ACA CAC GC	181
Prdx-1_F<br>Prdx-1_R	TGT CTT GTC CTG CTC CCA CA<br>GAC ATT CAG AAA GAG CCC CGT C	182
CYP_F<br>CYP_R	TCG AAG TCC TTG TTT CGA GTT GAG<br>TGG TGT CGA TGT GGA TTA TGC TG	173
GST_F<br>GST_R	AAT CCA GGT GCT TCC CAC AC<br>CAC GTC ATT TGT ATG GCC AAG TGA	158
